# Effect of secondary electron generation on dose enhancement in Lipiodol with and without a flattening filter

**DOI:** 10.1002/acm2.12282

**Published:** 2018-02-15

**Authors:** Daisuke Kawahara, Shuichi Ozawa, Akito Saito, Tomoki Kimura, Tatsuhiko Suzuki, Masato Tsuneda, Sodai Tanaka, Takeo Nakashima, Yoshimi Ohno, Yuji Murakami, Yasushi Nagata

**Affiliations:** ^1^ Radiation Therapy Section Department of Clinical Support Hiroshima University Hospital Hiroshima Japan; ^2^ Medical and Dental Sciences Course Graduate School of Biomedical & Health Sciences Hiroshima University Hiroshima Japan; ^3^ Department of Radiation Oncology Institute of Biomedical & Health Sciences Hiroshima University Hiroshima Japan; ^4^ Hiroshima High‐Precision Radiotherapy Cancer Center Hiroshima Japan; ^5^ Department of Nuclear Engineering and Management School of Engineering University of Tokyo Tokyo Japan; ^6^ Tokyo Women's Medical University Graduate School of Medicine Medical Physics Tokyo Japan

**Keywords:** energy spectrum, Lipiodol, Monte Carlo calculation, PEG

## Abstract

**Purpose:**

Lipiodol, which was used in transcatheter arterial chemoembolization before liver stereotactic body radiation therapy (SBRT), remains in SBRT. Previous we reported the dose enhancement in Lipiodol using 10 MV (10×) FFF beam. In this study, we compared the dose enhancement in Lipiodol and evaluated the probability of electron generation (PEG) for the dose enhancement using flattening filter (FF) and flattening filter free (FFF) beams.

**Methods:**

FF and FFF for 6 MV (6×) and 10× beams were delivered by TrueBeam. The dose enhancement factor (DEF), energy spectrum, and PEG was calculated using Monte Carlo (MC) code BEAMnrc and heavy ion transport code system (PHITS).

**Results:**

DEFs for FF and FFF 6× beams were 7.0% and 17.0% at the center of Lipiodol (depth, 6.5 cm). DEFs for FF and FFF 10× beams were 8.2% and 10.5% at the center of Lipiodol. Spectral analysis revealed that the FFF beams contained more low‐energy (0–0.3 MeV) electrons than the FF beams, and the FF beams contained more high‐energy (>0.3 MeV) electrons than the FFF beams in Lipiodol. The difference between FFF and FF beam DEFs was larger for 6× than for 10×. This occurred because the 10× beams contained more high‐energy electrons. The PEGs for photoelectric absorption and Compton scattering for the FFF beams were higher than those for the FF beams. The PEG for the photoelectric absorption was higher than that for Compton scattering.

**Conclusions:**

FFF beam contained more low‐energy photons and it contributed to the dose enhancement. Energy spectra and PEGs are useful for analyzing the mechanisms of dose enhancement.

## 1 INTRODUCTION

In stereotactic body radiation therapy (SBRT) of liver cancer, high‐dose radiation delivered using hypo‐fractionation increases the probability of tumor control and patient survival.^1^, ^2^ Accurate daily localization of the treatment target is very important owing to a large irradiation dose delivered in a short period of time.^3^, ^4^

In liver SBRT, Lipiodol has been used as an embolic agent and for tumor seeking in transarterial chemoembolization (TACE). Lipiodol is ethiodized oil, is a poppyseed oil used by injection as a radio‐opaque contrast agent. Several institutions have recently reported promising responses in patients with unrespectable hepatocellular carcinoma treated with TACE followed by radiation therapy.^5^, ^6^ Our previous study reported that MC calculation demonstrated a large dose enhancement in the Lipiodol region using a virtual phantom and clinical patient CT.^7^ However, we only used 10 MV (10×) flattening filter free (FFF) beam and the factor of the dose enhancement was not revealed.

Recently, medical linear accelerators capable of generating flattening filter free (FFF) beams were developed. FFF beam offer increasing the dose delivery efficiency of state of the art radiotherapy techniques such as intensity modulated radiotherapy and SBRT.^8^, ^9^ While FFF beams are advantageous for radiation dose delivery, the removal of the flattening filter largely decreases the beam attenuation and increases the photon spectrum. Thus, it also affects the photon energy distribution or the beam quality.^10^, ^11^ For a FFF beam, these low‐energy photons are part of the beam and contribute to the dose deposition in the photon beam buildup region close to the patient's body surface. Compared with FF beams, FFF beams exhibit less head scatter and leakage; however, measurements and Monte Carlo (MC) simulations suggest that irradiation by FFF beams results in higher surface doses compared with FF beams.^12^, ^13^ This suggests that low‐energy photons may play an important role in the surface dose enhancement by FFF beams. Therefore, there is a possible that the difference of the energy spectrum such as FF and FFF beams also affect the dose enhancement.

In this study, we compared the dose enhancements in Lipiodol and evaluated the probability of electron generation (PEG) which present the factor of the dose enhancement, for FF and FFF beams.

## 2 METHODS AND MATERIALS

### 2.A MC calculations

A TrueBeam linear accelerator (Varian Medical Systems, Palo Alto, USA) that generated FFF and FF beams with 6 MV (6×) and 10× was used in this study. The MC code BEAMnrc was used to model the TrueBeam linear accelerator (linac).^14^, ^15^, ^16^, ^17^ The components of the TrueBeam accelerator's head are proprietary and not available to the public for direct simulations; however, Varian provides IAEA‐compliant phase‐space files, which were simulated using the GEANT4 MC code, located just above the secondary X/Y collimator. The phase space was scored onto the surface of a cylinder located above the secondary collimator. Therefore, the phase‐space files below the secondary collimator were modeled using Beamnrc. The phase‐space data scored at a source‐to‐surface distance (SSD) of 70 cm were used as input data for an inhomogeneity virtual phantom. Dose calculation, and photon and electron energy spectra acquisitions were performed using the MC code PHITS. Although BEAMnrc can easily create the linac model, BEAMnrc cannot analyze the energy spectrum in the phantom. PHITS can deal with the transport of nearly all particles, including neutrons, protons, heavy ions, photons, and electrons, over wide energy ranges using several nuclear reaction models and nuclear data libraries.^18^ The dose calculation grid size was 2.0 mm. The cutoff energies for photons and electrons were set to 0.01 MeV. The number of photon histories in Beamnrc and PHITS was 2.0 × 10^8^ and 2.0 × 10^9^, respectively. In our previous study, the MC calculation accuracy was validated by comparing its results with the percent depth dose (PDD) and off axis ratio measured with water equivalent phantom that contained Lipiodol.^7^ It showed that the measured data and the MC results agreed within 3%.

### 2.B DEF and the energy spectral variations of photons and electrons for a virtual phantom

**Figure 1:**
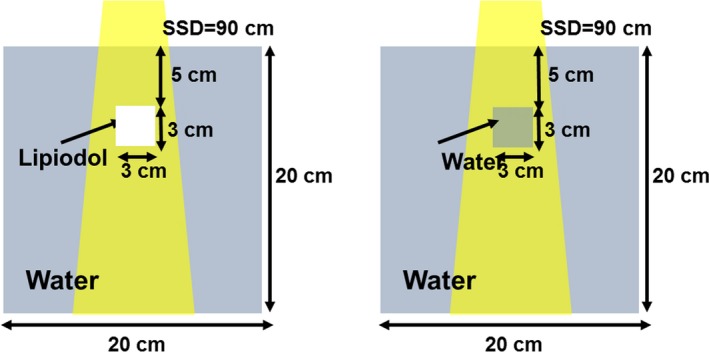
A geometric scheme of Lipiodol was located at depth of 5.0 cm in a water‐equivalent phantom (20 × 20 × 20 cm^3^).

The dose enhancement factor (DEF) was defined as a ratio of the average deposited dose to the volume, both with and without the presence of Lipiodol, after the MC simulation. We considered a virtual inhomogeneity phantom, with Lipiodol (3 × 3 × 3 cm^3^) located at a depth of 5.0 cm in a water‐equivalent phantom (20 × 20 × 20 cm^3^) (Fig. 1). The size of the Lipiodol was determined by the report of John, et al.^19^ They measured the size of the liver tumor size and it was approximately 3 cm. A 5 × 5 cm^2^ field was used for irradiating at the SSD = 90 cm. The PDD was measured and normalized to the calculated dose at *D*_max_. Lipiodol, ethiodized oil injection, is a sterile injectable radio‐opaque diagnostic agent. Each milliliter contains 480 mg of Iodine organically combined with ethyl esters of fatty acids of poppyseed oil. The mass density of Lipiodol was set to 1.28 g/cm^3^.

**Figure 2:**
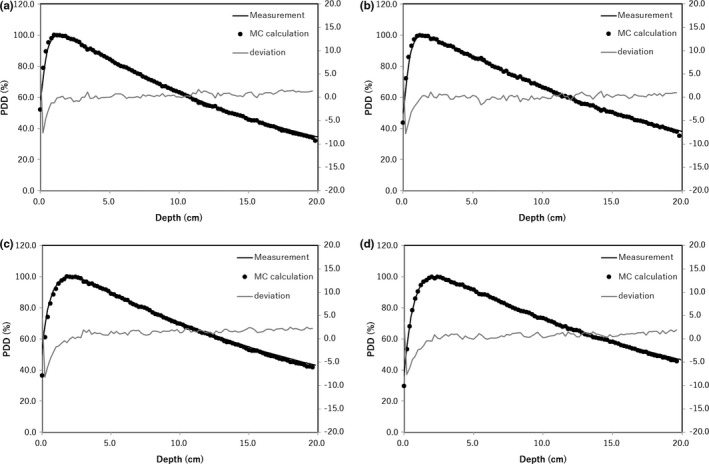
The validation of PDD curves comparing the film measurement with MC calculation with FFF beam (left) and FF beam (right) for 6× and 10×.

The energy spectral variations of photons and electrons were investigated using the same beam and virtual phantom (this section). The number of bins in each spectrum was set to 50, with energy ranging from 0 MeV to 20 MeV. The energy spectrum was analyzed at 6.5 cm depth that was the center of the Lipiodol with and without the Lipiodol and the energy spectrum was normalized at the dose per MU with FF and FFF beams for 6× and 10× (Fig. 2).

### 2.C The probability of electron generation

PEG was defined as the probability of the secondary electron generation. The PEG was calculated with the number of photons in an energy bin and the ratio of the energy absorption coefficient to the mass attenuation coefficient. The photon energy spectrum was different between FF and FFF for both 6× and 10×. In addition, the photon energy was varied with location and material. The photon interaction coefficient depends on the energy spectrum. For photon interactions, we considered only the photoelectric effect and Compton scattering, because these processes are predominant at energies below 10 MeV in the water and the Lipiodol. The absorption probability for the photon interactions and energy absorption coefficient data were taken from Berger et al.^20^ The PEGs with the photoelectric effect (PEG_PE_) and Compton effect (PEG_CE_) were calculated a rate of photon energy presence multiplied by the energy absorption coefficient and absorption probability of photon interactions. The total of PEG (PEG_Total_) was defiend as sum of the PEG with the photoelectric effect and that with Compton effect.

## 3 RESULTS

### 3.A DEF and the energy spectral variations of photons and electrons, for the virtual phantom

**Figure 3:**
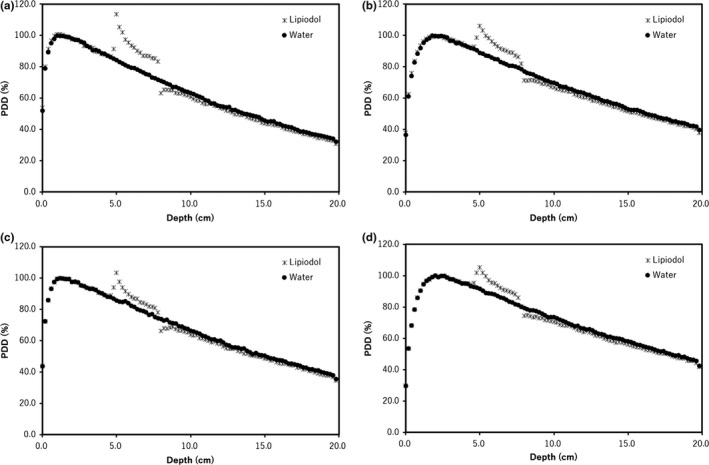
MC‐calculated PDD curves for the virtual phantoms with (asterisk) and without (closed circles) Lipiodol with (a) 6× FFF, (b) 6× FF, (c) 10× FFF and (d) 10× FF beams.

**Figure 4:**
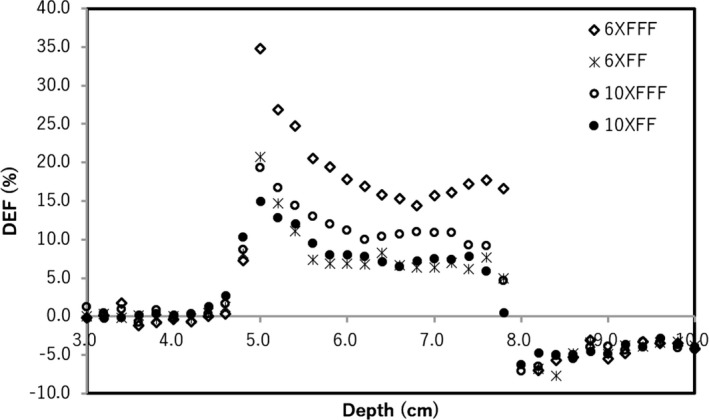
The dose enhancement ratio with the virtual water phantom which Lipiodol was located at depth of 5.0 cm for 6× FFF (open diamond), 6× FF (asterisk), 10× FFF (open circle), and 10× FF (closed circle) beams.

Figure 3 shows the PDD curves calculated from MC simulations for the water phantom with and without Lipiodol, using the FF and FFF beams for 6× and 10×. All PDDs were normalized to 100% at *d*_max_ for each beam. Figure 4 shows the DEFs with FF and FFF beams for 6× and 10×. The DEFs with FF and FFF beams for 6× were 7.0% and 17.0% at the center of Lipiodol. The DEFs with FF and FFF beams for 10× beams were 8.2% and 10.5% at the center of Lipiodol. The DEF deviation across the FFF and FF beams for 6× was larger than 10×.

**Figure 5:**
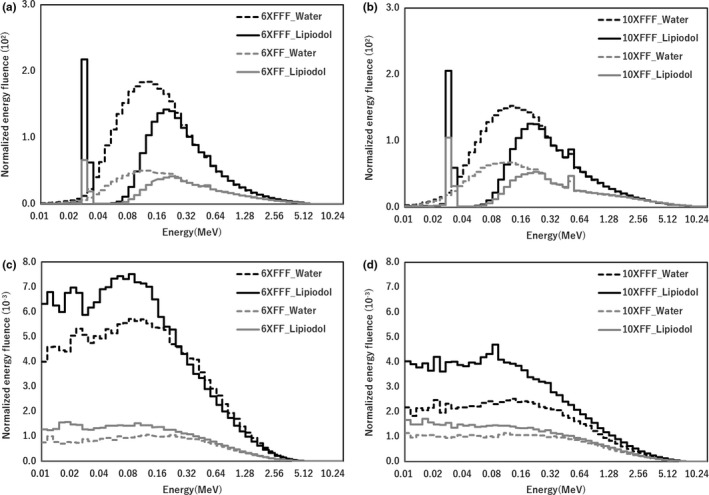
The disrubutions of photon energy spectrum and energy at the center of Lipiodol (depth, 6.5 cm), for irradiation with FFF and FF beams with 6× and 10×. (a, b) Photon spectra for irradiation with 6× and 10× beams, respectively. (c, d) Electron spectra for irradiation with 6× and 10× beams, respectively.

Figure 5 shows the distributions of photon energy spectrum with FF and FFF beams for 6× and 10×. The energy spectra of photon and electrons were normalized to the maximum intensity. In the Lipiodol, the photon energy spectra contained the peak in the 0.03–0.04 MeV range. Compared with FF beam, FFF beam contained more photons with energies mostly in the 0–1.5 MeV range for 6×. Compared with FF beam, FFF beam contained more photons, with energies mostly in the 0–1.5 MeV range for 10×. Moreover, the FFF beams featured more low‐energy (0–0.3 MeV) electrons than the FF beams for both 6× and 10×.

### 3.B Evaluation of the PEG

**Figure 6:**
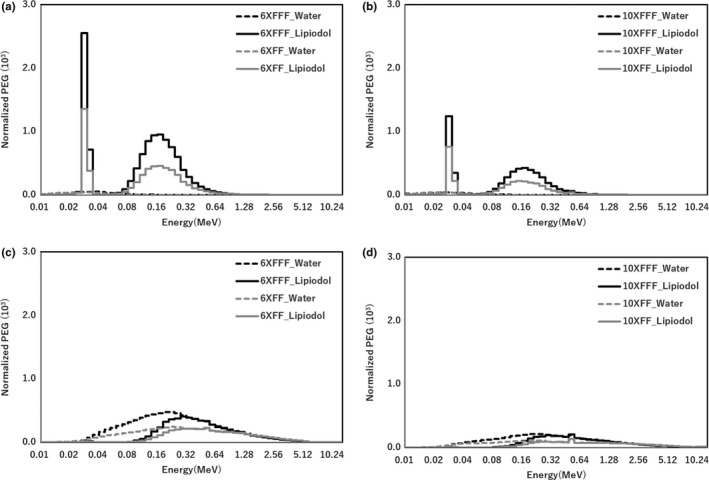
PEG at the center of the Lipiodol (6.5 cm depth) with FFF and FF for 6× and 10× beams. (a, b) PEG by photoelectric effect for 6× and 10×, respectively. (c, d) PEG by Compton scattering for 6× and 10×, respectively.

**Figure 7:**
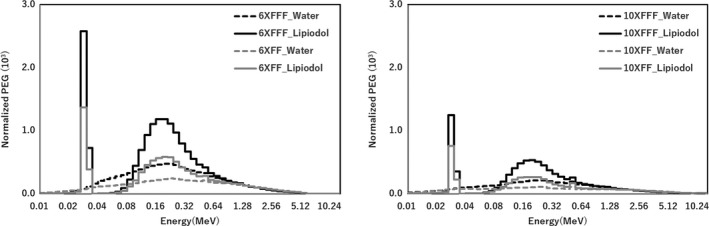
Total PEG at the center of the Lipiodol (6.5 cm depth) with FFF and FF for 6× and 10× beams.

Figure 6 shows that the PEG owing to the photoelectric effect was larger than that owing to the Compton scattering with FF and FFF beams for 6× and 10× in the Lipiodol. The PEGs were normalized to the maximum intensity. The PEG owing to the Compton scattering was dominant compared to that owing to the photoelectric effect, and PEG owing to the Compton scattering increased for energies in the 0.02–5.0 MeV range in the water. The PEG owing to the photoelectric effect was maximal at 0.03–0.04 MeV range in Lipiodol and increased for energies in the 0.06–0.6 MeV range. The PEG owing to the Compton scattering increased for energies in the 0.1–5.0 MeV range, in Lipiodol. Figure 7 shows the total PEG with FF and FFF beams for 6× and 10×. Comparing the results for the FF and FFF beams, the total PEG for the FFF beam was larger than that FF beam (<1.5 MeV) for 6×, and the total PEG for the FFF beam was larger than that FF beam (<2.5 Mev) for 10× with and without Lipiodol.

## 4 DISCUSSION

In a previous study, Alkhatib et al. considered the probability of pair production in high‐atomic‐number materials. They concluded that it was important to understand the interactions better.^21^ Alkhatib et al. demonstrated the local dose enhancement in and around “high Z” materials, but they only reported electron‐positron pairs as a function of the kinetic energy. In our study, we evaluated the dose enhancement in Lipiodol and analyzed the contribution of PEG and energy spectra of photons and electrons to the dose enhancement.

There are two factors of the dose enhancement in the Lipiodol. One is the iodine K‐edge of 33.2 keV, which was contained the photon energy spectra in the Lipiodol from the result of Fig. 5. As shown in Fig. 6, the PEG was also high value at 0.03–0.04 MeV range. Thus, the iodine K‐edge of 33.2 keV contributes the dose enhancement. The other is dependence of the photon cross‐section on the material composition. Berger et al. showed that the energy at the boundary between the photoelectric effect and Compton scattering was higher for Lipiodol (0.3 MeV) than for water (0.03 MeV). Therefore, the PEG owing to the Compton scattering was higher than that owing to the photoelectric effect in the case of water, while the PEG owing to the photoelectric effect was higher than that owing to the Compton scattering in the Lipiodol. From the results in Fig. 7, the total PEG for Lipiodol was higher than that for water. Thus, it is concluded that the PEG owing to the photoelectric effect is a primary determinant of the dose enhancement.

A previous study investigated the surface dose difference across FFF and FF beams.^12^ That study reported that FFF beams yield a modestly higher surface dose in the buildup region compared with FF beams for field sizes ≤10 × 10 cm^2^. In addition, FFF beams contained more low‐energy photons in the buildup region. In our study, the same trend was observed; the FFF beams contained more low‐energy photons (0–0.3 MeV) at a depth of 8.5 cm in water, as shown in Fig. 5. Thus, the PEG owing to the photoelectric effect and Compton scattering was higher at low energies, and it was dominant for the total PEG. Moreover, the DEF deviation with FF and FFF beams for 6× was larger than 10× beams. This also occurred because the 6× beams contained more low‐energy photons than the 10× beams, which predominantly determined the total PEG. There were large deviations of FF and FFF beams in the electron energy range ≤ 500 keV form the result of Figs. 5(c) and 5(d). Wayne D Newhauser, et al. reported that the electron energy range at 500 keV in the water is approximately 2 mm.^22^ The electron range in the Lipiodol is shorter than the water because the Lipiodol is high density and high Z material. Energy spectra and PEG are useful for analyzing the mechanisms of dose enhancement in the dose calculation grid. The presence of low‐energy photons was a stronger determinant of PEG, which contributed to the dose enhancement.

However, it was the limitation of this study for the estimation of the concentration of Lipiodol from clinical patient CT. For clinical patient use, the assignment of material type according to the density of Lipiodol is now underway with dual energy CT. Moreover, we do not consider various clinical conditions such as the effect of multi leaf collimator (MLC), field size and more. The past study reported the difference of output factor with FF and FFF beams was within 2% and the difference of the total scatter factor with and without MLC was within 1% for small field.^23^, ^24^

We revealed the factor of the dose enhancement with the analysis of energy spectrum and PEG with FF and FFF beams for 6× and 10×. DEF was larger with FF beam than FFF beam in the Lipiodol. FFF beam shortened beam delivery time and it contribute to higher dose enhancement. Considering the balance of tumor coverage and skin sparing, there is a possibility that FFF beam would be useful for liver SBRT who Lipiodol was remained in the tumor.

## 5 CONCLUSION

The difference of DEFs with FF and FFF were 10.0% and 2.3% at the center of Lipiodol for 6× and 10×, respectively. The photons at the 33.2 keV and the 0.06–1.5 MeV ranges for 6 MV, and the 33.2 keV and the 0.06–2.5 MeV ranges for 10 MV contribute the dose enhancement. FFF beam contained more low‐energy photons and it contributed to the dose enhancement. Energy spectra and PEGs are useful for analyzing the mechanisms of dose enhancement.

## CONFLICT OF INTEREST

None.
